# Clinical and Imaging Characteristics, Care Pathways, and Outcomes of Traumatic Epidural Hematomas: A Collaborative European NeuroTrauma Effectiveness Research in Traumatic Brain Injury Study

**DOI:** 10.1227/neu.0000000000002982

**Published:** 2024-05-21

**Authors:** Dana Pisică, Victor Volovici, John K. Yue, Thomas A. van Essen, Hugo F. den Boogert, Thijs Vande Vyvere, Iain Haitsma, Daan Nieboer, Amy J. Markowitz, Esther L. Yuh, Ewout W. Steyerberg, Wilco C. Peul, Clemens M. F. Dirven, David K. Menon, Geoffrey T. Manley, Andrew I. R. Maas, Hester F. Lingsma

**Affiliations:** * Department of Public Health, Center for Medical Decision Making, Erasmus MC - University Medical Center Rotterdam, Rotterdam, the Netherlands;; ‡ Department of Neurosurgery, Erasmus MC - University Medical Center Rotterdam, Rotterdam, the Netherlands;; § Department of Neurosurgery, University of California, San Francisco, San Francisco, California, USA;; ‖ Brain and Spinal Injury Center, Zuckerberg San Francisco General Hospital, San Francisco, California, USA;; ¶ University Neurosurgical Center Holland, Leiden University Medical Center, Haaglanden Medical Center and Haga Teaching Hospital, Leiden and The Hague, the Netherlands;; # Department of Neurosurgery, Leiden University Medical Center, Leiden, the Netherlands;; ** Division of Neurosurgery, Department of Surgery, QEII Health Sciences Centre and Dalhousie University, Halifax, Nova Scotia, Canada;; †† Department of Neurosurgery, Radboud University Medical Center, Nijmegen, the Netherlands;; ‡‡ Department of Radiology, Antwerp University Hospital and University of Antwerp, Antwerp, Belgium;; §§ Department of Radiology and Biomedical Imaging, University of California, San Francisco, San Francisco, California, USA;; ‖‖ Department of Biomedical Data Sciences, Leiden University Medical Center and Haaglanden Medical Center, Leiden and The Hague, the Netherlands;; ¶¶ Division of Anaesthesia, University of Cambridge and Addenbrooke's Hospital, Cambridge, UK;; ## Department of Neurosurgery, Antwerp University Hospital, Edegem, Belgium;; *** Department of Translational Neuroscience, Faculty of Medicine and Health Science, University of Antwerp, Antwerp, Belgium

**Keywords:** Clinical decision-making, Cranial epidural hematoma, Guideline, Neurosurgical procedure, Traumatic brain injury

## Abstract

**BACKGROUND AND OBJECTIVES:**

Guideline recommendations for surgical management of traumatic epidural hematomas (EDHs) do not directly address EDHs that co-occur with other intracranial hematomas; the relative rates of isolated vs nonisolated EDHs and guideline adherence are unknown. We describe characteristics of a contemporary cohort of patients with EDHs and identify factors influencing acute surgery.

**METHODS:**

This research was conducted within the longitudinal, observational Collaborative European NeuroTrauma Effectiveness Research in Traumatic Brain Injury cohort study which prospectively enrolled patients with traumatic brain injury from 65 hospitals in 18 European countries from 2014 to 2017. All patients with EDH on the first scan were included. We describe clinical, imaging, management, and outcome characteristics and assess associations between site and baseline characteristics and acute EDH surgery, using regression modeling.

**RESULTS:**

In 461 patients with EDH, median age was 41 years (IQR 24-56), 76% were male, and median EDH volume was 5 cm^3^ (IQR 2-20). Concomitant acute subdural hematomas (ASDHs) and/or intraparenchymal hemorrhages were present in 328/461 patients (71%). Acute surgery was performed in 99/461 patients (21%), including 70/86 with EDH volume ≥30 cm^3^ (81%). Larger EDH volumes (odds ratio [OR] 1.19 [95% CI 1.14-1.24] per cm^3^ below 30 cm^3^), smaller ASDH volumes (OR 0.93 [95% CI 0.88-0.97] per cm^3^), and midline shift (OR 6.63 [95% CI 1.99-22.15]) were associated with acute surgery; between-site variation was observed (median OR 2.08 [95% CI 1.01-3.48]). Six-month Glasgow Outcome Scale–Extended scores ≥5 occurred in 289/389 patients (74%); 41/389 (11%) died.

**CONCLUSION:**

Isolated EDHs are relatively infrequent, and two-thirds of patients harbor concomitant ASDHs and/or intraparenchymal hemorrhages. EDHs ≥30 cm^3^ are generally evacuated early, adhering to Brain Trauma Foundation guidelines. For heterogeneous intracranial pathology, surgical decision-making is related to clinical status and overall lesion burden. Further research should examine the optimal surgical management of EDH with concomitant lesions in traumatic brain injury, to inform updated guidelines.

**ABBREVIATIONS:**
ASDHs: acute subdural hematomas
BTF: Brain Trauma Foundation
CENTER-TBI: Collaborative European NeuroTrauma Effectiveness Research in TBI
EDHs: epidural hematomas
GOSE: GOS-Extended
IPHs: intraparenchymal hemorrhages
MOR: median OR
MLS: midline shift
TBI: traumatic brain injury
TSAH: traumatic SAH.

Recent studies estimate that traumatic epidural hematomas (EDHs) are present in 8% to 19% of patients with traumatic brain injury (TBI).^1-3^ EDHs are believed to portend better prognosis compared with other types of traumatic intracranial mass lesions, such as acute subdural hematomas (ASDHs) and intraparenchymal hemorrhages (IPHs).^4-9^ This is potentially because of the absence of direct parenchymal damage and/or, in the case of voluminous EDHs, the efficacy of early evacuation.^5,10-13^

The 2006 Brain Trauma Foundation (BTF) guidelines on surgical management of EDHs recommend evacuation of all EDHs ≥30 cm^3^, regardless of Glasgow Coma Scale (GCS) score and specify clinical and radiographic parameter thresholds for nonoperative management with close neurological observation and serial scanning.^14^ A substantial portion of EDH literature, including most of the BTF guideline evidence base, is based on relatively small, retrospective, highly selected patient samples (eg, restricted to EDHs with no or “minor” concomitant hematomas; severe TBI; exclusively surgical cohorts). Consequently, knowledge of the entire EDH spectrum and distribution of clinical presentations, imaging phenotypes, management strategies, and outcomes is limited. Particularly, the prevalence of nonisolated EDHs and the influence of concomitant intracranial hematomas on surgical decision-making are unknown. Guideline adherence in current practice, especially given changing TBI demographics,^15-20^ has not been evaluated.

In the setting of the large, prospective, multicenter, observational Collaborative European NeuroTrauma Effectiveness Research in TBI (CENTER-TBI) core study, we aimed to describe clinical and imaging characteristics, management pathways, BTF guideline adherence, and outcomes of patients with EDHs. Characteristics were compared between patients with isolated EDH and those with concomitant ASDH and/or IPH. We additionally aimed to identify site and baseline factors influencing acute surgery targeting either the EDH itself or overall mass lesions.

## METHODS

This study follows Strengthening the Reporting of Observational Studies in Epidemiology statement recommendations.^21^

### Study Design

This study was performed within the CENTER-TBI core study (ClinicalTrials.gov—NCT02210221; Resource Identification Portal—Research Resource Identifier: SCR_015582), a longitudinal, observational cohort study that prospectively enrolled patients from 65 study sites in 18 countries across Europe and Israel from December 2014 to December 2017.

### Study Population

CENTER-TBI enrolled patients within 24 hours of injury, with a clinical TBI diagnosis, a head computed tomography (CT) scan indication, and no severe pre-existing neurologic disorders that could confound outcome assessment, and has been described previously.^22^ Ethical approval was obtained by each site; written informed consent was provided by all enrolled participants/legal representatives/next of kin (**Supplemental Digital Content 1**, http://links.lww.com/NEU/E227).

For this analysis, we selected CENTER-TBI participants across the severity spectrum with at least 1 EDH of any volume on the first CT scan, performed at the study site within 36 hours of injury.

### Data Collection and Management

Data collection, handling, and storage were detailed previously.^16,22^ Data were extracted using an internal retrieval interface (Neurobot version 3.0, International Neuroinformatics Coordinating Facility; https://center-tbi.incf.org/; data freeze December 2022). Patient selection was based on structured reports of available and interpretable first scans, reviewed centrally according to TBI radiologic Common Data Elements.^23,24^ Hematoma volumes were calculated using the width × depth × length × 0.5 formula. When multiple hematomas were present, total volume was calculated by adding individual lesion volumes, separately for each hematoma type. Large EDH was defined as total volume ≥30 cm^3^, the BTF guideline threshold for evacuation.^14^ We defined isolated EDH as no concomitant ASDH and/or IPH, but potentially concomitant traumatic subarachnoid hemorrhage (TSAH). Nonisolated EDH was defined by at least one concomitant ASDH and/or IPH, regardless of size.

### Interventions

Preferred local treatment strategies for management of intracranial hematomas were followed, permitting analysis of contemporary practice and guideline adherence (**Supplemental Digital Content 1**, http://links.lww.com/NEU/E227).

“Early targeted EDH evacuation” was defined as any intervention scheduled and conducted after the first scan (ie, early) in which EDH evacuation was the main indication (ie, targeted), potentially with simultaneous evacuation of adjacent ASDHs and/or IPHs. The alternative was any early EDH management course, other than targeted evacuation, and could include the following scenarios: (1) early nontargeted evacuation of an (usually small) EDH during interventions for other indications (eg, adjacent ASDH); (2) early ASDH and/or IPH evacuation/decompression, without EDH evacuation; (3) minor early cranial surgery, without any hematoma evacuation (eg, debridement); (4) initial observation and/or repeat scanning, potentially followed by delayed cranial surgery.

“Any early hematoma evacuation” was defined as any intervention scheduled and conducted after the first scan in which any hematoma (EDH and/or ASDH and/or IPH) was evacuated/decompressed, regardless of which lesion constituted the main indication. This included early targeted EDH evacuation and scenarios (1) and (2) above. The alternative, initial conservative management of overall mass lesions, was defined as scenarios (3) or (4) above.

### Outcomes

For descriptive analyses, the primary outcome was Glasgow Outcome Scale–Extended (GOSE) score at 6 months postinjury (**Supplemental Digital Content 1**, http://links.lww.com/NEU/E227).^25,26^ Secondary outcomes included hospital and intensive care unit (ICU) lengths of stay and in-hospital mortality.

### Statistical Analyses

Clinical, imaging, management, and outcome data were reported as absolute and relative frequencies for categorical variables and medians and IQR for continuous variables. Characteristics are reported for the entire sample and separately for participants with isolated and nonisolated EDHs. Differences between subgroups were tested using Mann-Whitney *U* tests and χ^2^ statistics.

Management pathways were visualized using Sankey diagrams, depicting participant trajectories across 3 stages: (1) total EDH volume on first scan, (2) early clinical course after first scan, and (3) entire clinical course, including delayed/follow-up interventions.^27^ Diagrams were created for the entire sample and subgroups: isolated, nonisolated EDH, and according to the BTF guideline recommendation categories (**Supplemental Digital Content 1**, http://links.lww.com/NEU/E227).

Fixed-effects logistic regression was performed to assess associations between baseline clinical and imaging variables and the 2 acute interventions. Independent variables were selected based on previous literature^14^ and expert opinion. Case-mix adjusted between-site variation in acute interventions was quantified for sites enrolling >10 participants with the median odds ratio (MOR). Modeling details, including handling nonlinearity, the definition and calculation of the MOR, are further described in **Supplemental Digital Content 1** (http://links.lww.com/NEU/E227).

Variable associations with the interventions were reported as ORs and 95% CIs. The proportion of explained “variation” in interventions was calculated using Nagelkerke pseudo-R^2^.

Multiple imputation^28^ based on a large number of variables was used for missing regression covariates. Analyses were performed using R^29^ version 4.0.3 (R Foundation for Statistical Computing) and RStudio^30^ version 2022.7.1.554 (RStudio, PBC); 2-sided *P* < .05 was considered statistically significant.

## RESULTS

CENTER-TBI enrolled 4509 participants, of whom 4068 had an available and interpretable first scan within 36 hours of injury. Of these, 461 participants (11% of all TBI, 16% of severe TBI) from 53 enrolling sites sustained EDHs.

Median age of participants was 41 years (IQR 24-56); 348 (76%) were male. The most frequent cause of injury was a fall (43%). The majority presented with a GCS score 13 to 15 (52%) and both pupils reactive (86%) (Table 1).

**TABLE 1.** Baseline Clinical and Imaging Characteristics of All Participants With EDHs and by Presence of Concomitant ASDHs and/or IPHs on the First Scan

Characteristic	Total (n = 461)	Findings on the first scan		*P* value^a^	Missing values (%)
		Isolated EDH (n = 133)	Nonisolated EDH (n = 328)		
Clinical					
Age, median [IQR], y	41 [24, 56]	32 [21, 50]	44 [29, 57]	<.001	0.0
Male sex (%)	348 (75.5)	101 (75.9)	247 (75.3)	.98	0.0
Injury cause (%)				.10	4.6
Road traffic incident	160 (36.4)	47 (36.2)	113 (36.5)		
Incidental fall	191 (43.4)	49 (37.7)	142 (45.8)		
Other	89 (20.2)	34 (26.2)	55 (17.7)		
Baseline GCS score^b^, median [IQR]	13 [7, 15]	15 [12, 15]	11 [5, 14]	<.001	4.6
Baseline GCS-Motor score^b^, median [IQR]	6 [3, 6]	6 [6, 6]	5 [1, 6]	<.001	2.2
TBI severity (%)				<.001	4.6
Mild (Baseline GCS score^b^ ≥13)	227 (51.6)	97 (74.6)	130 (41.9)		
Moderate (Baseline GCS score^b^ 9-12)	76 (17.3)	14 (10.8)	62 (20.0)		
Severe (Baseline GCS score^b^ ≤8)	137 (31.1)	19 (14.6)	118 (38.1)		
One/both unreactive pupils at baseline^b^ (%)	59 (13.6)	10 (8.2)	49 (15.7)	.06	5.9
Focal neurologic deficit (%)	46 (12.8)	7 (5.9)	39 (16.2)	.01	22.1
Lucid interval (%)	29 (8.5)	9 (8.8)	20 (8.3)	1.00	25.8
Major extracranial injury^c^ (%)	215 (46.6)	60 (45.1)	155 (47.3)	.75	0.0
Imaging					
Time from injury to first scan, median [IQR], h	2 [1, 3]	2 [1, 3]	2 [1, 3]	.25	0.0
EDH volume^d^, median [IQR], cm^3^	5 [2, 20]	4 [2, 13]	6 [2, 22]	.07	0.0
Large EDH (EDH volume^d^ ≥30 cm^3^)	86 (18.7)	19 (14.3)	67 (20.4)	.16	0.0
Multiple EDHs (%)	103 (22.3)	20 (15.0)	83 (25.3)	.02	0.0
EDH in temporal region (%)	265 (57.5)	66 (49.6)	199 (60.7)	.04	0.0
EDH in frontal region (%)	174 (37.7)	57 (42.9)	117 (35.7)	.18	0.0
Skull fracture (%)	452 (98.0)	133 (100.0)	319 (97.3)	.12	0.0
ASDH (%)	197 (42.7)	NA	197 (60.1)	NA	0.0
IPH (%)	292 (63.3)	NA	292 (89.0)	NA	0.0
Traumatic subarachnoid hemorrhage (%)	333 (72.2)	60 (45.1)	273 (83.2)	<.001	0.0
Midline shift (%)	95 (20.6)	14 (10.5)	81 (24.7)	.001	0.0
Cisternal compression (%)	156 (33.8)	20 (15.0)	136 (41.5)	<.001	0.0

ASDH, acute subdural hematoma; EDH, epidural hematoma; GCS, Glasgow Coma Scale; IPH, intraparenchymal hemorrhage; NA, not applicable; TBI, traumatic brain injury.

a *P* values derived from χ^2^ statistics for categorical variables and Mann-Whitney *U* tests for continuous variables (all non-normally distributed), comparing the isolated and nonisolated EDH subgroups. The *P* value assessed the compatibility with the null hypothesis of no differences between the 2 subgroups.

b When possible, missing values were imputed using International Mission for Prognosis and Analysis of Clinical Trials in TBI methodology: Take poststabilization value and if absent, work back in time toward prehospital values until nonmissing value is found.

c Any extracranial injury with an Abbreviated Injury Scale score ≥3.

d Volumes of individual lesions were estimated using the width × depth × length × 0.5 formula. When multiple EDHs were present simultaneously, their volumes were added up.

**FIGURE 1.**
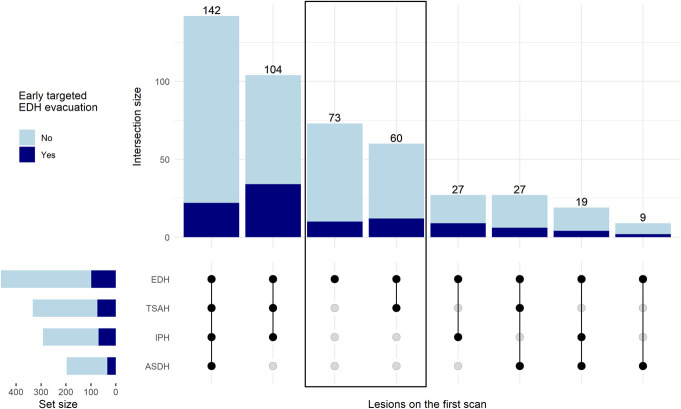
Concomitant radiologic findings on the first scan in participants with EDHs. The boxed area represents the subgroup we defined as isolated (133 participants), meaning no concomitant ASDHs and/or IPHs, which are space-occupying lesions that may warrant surgical intervention on their own. By this definition, isolated EDHs included cases with coexisting TSAH but also with other intracranial traumatic findings not represented here, such as intraventricular hemorrhage and traumatic axonal injury, meaning that radiologic “pure” EDHs are even more infrequent. Abbreviations: ASDH, acute subdural hematoma; EDH, epidural hematoma; IPH, intraparenchymal hemorrhage; TSAH, traumatic subarachnoid hemorrhage.

Isolated EDH occurred in 133 participants (29%), 60 of which had concomitant TSAH. At least one concomitant ASDH (43%) and/or IPH (63%) was present in 328 participants (71%). The most frequent radiographic phenotype was all 4 hemorrhage types simultaneously present (142 participants, 31%) (Figure 1). Median total EDH volume was 5 cm^3^ (IQR 2-20), with 86 participants (19%) having large EDHs ≥30 cm^3^. EDHs extended most frequently temporally (58%) and frontally (38%) (Table 1, **Supplemental Digital Content 2** [http://links.lww.com/NEU/E228]).

**FIGURE 2.**
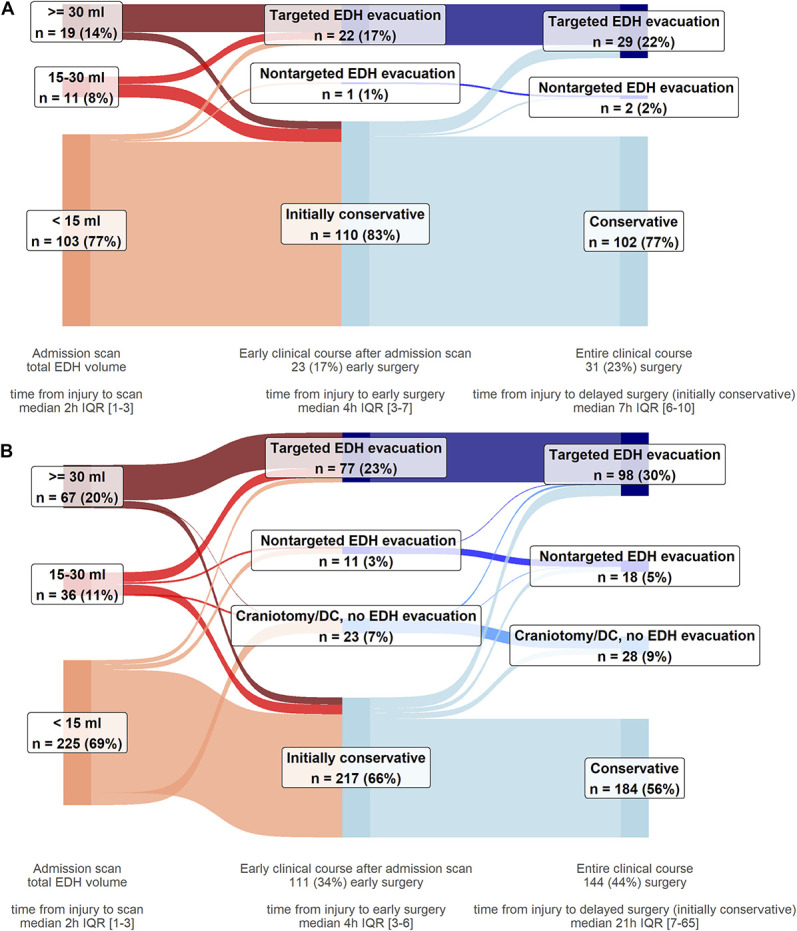
Surgical care pathways of participants with EDHs, by presence of concomitant acute subdural hematomas and/or IPHs on the first scan. **A**, Participants with isolated EDHs (n = 133). Most of the isolated EDHs had volumes <15 cm^3^ (103 participants, 77%), most of which were treated conservatively throughout the entire clinical course (96 participants, 93%). Participants with isolated EDHs between 15 and 30 cm^3^ received either initially conservative treatment (7 participants, range EDH volumes 16-28 cm^3^) or early targeted EDH evacuation (4 participants, range EDH volumes 15-19 cm^3^, motivated by mass effect on computed tomography, or clinical deterioration). Most participants with isolated EDHs ≥30 cm^3^ received early targeted EDH evacuation (15 participants, 79%). Early targeted EDH evacuation was used after the first scan in 22 participants with isolated EDH (17%), with a median EDH volume of 54 cm^3^, IQR 18 to 110. Seven participants with isolated EDH who were not operated directly after the first scan (motivated by guideline adherence, little/no mass effect, no surgical lesion, or acceptable/good neurologic condition) later received delayed targeted EDH evacuation. In these, repeat scanning revealed hematoma enlargement, with a median EDH volume of 26 cm^3^ on the first scan and 44 cm^3^ on the last follow-up scan before surgery. Two participants received EDH evacuation during surgery for another indication, one in the early phase, for depressed skull fracture elevation (3 cm^3^ EDH), and the other during evacuation of a delayed large contusion. **B**, Participants with nonisolated EDHs (n = 328) Most of the nonisolated EDHs had volumes <15 cm^3^ (225 participants, 69%), most of which were treated conservatively throughout the entire clinical course (169 participants, 75%). Participants with nonisolated EDHs between 15 and 30 cm^3^ received either initially conservative treatment (42%, median EDH volume 21 cm^3^, IQR 17-22), early targeted EDH evacuation (42%, median EDH volume 23 cm^3^, IQR 21-26), or surgery for another main indication. Most participants with nonisolated EDHs ≥30 cm^3^ received early targeted EDH evacuation (55 participants, 82%). In the early clinical course, after the first scan, a third of participants with nonisolated EDH received a surgical intervention. Early targeted EDH evacuation was used in 77 participants (23%), with a median EDH volume of 46 cm^3^, IQR 29 to 78. Early nontargeted EDH evacuation was performed in 11 participants, during surgery for another main indication: ipsilateral ASDH evacuation or DC, elevation of adjacent depressed skull fracture. Early craniotomies/DCs for other indications, during which the EDH was not evacuated, were performed in 23 participants, who received contralateral ASDH evacuation/DC, supratentorial ASDH evacuation/DC (in participants with posterior fossa EDHs), ipsilateral ASDH evacuation/DC (EDH outside craniotomy window). Various delayed surgical interventions were used in 33 participants with nonisolated EDHs who initially received conservative management. Delayed targeted EDH evacuation was used in 21 participants, with a median EDH volume of 18 cm^3^ on the first scan and 44 cm^3^ on the last follow-up scan before surgery. ASDH, acute subdural hematoma; DC, decompressive craniectomy; EDH, epidural hematoma; IPH, intraparenchymal hemorrhage. Targeted EDH evacuation: EDH evacuation was the main surgical indication; Nontargeted EDH evacuation: EDH evacuation performed, but was not the main surgical indication; Craniotomy/DC, no EDH evacuation: craniotomy or DC for ASDH and/or IPH evacuation/decompression, during which the EDH was not evacuated; Initially conservative: initial nonsurgical management strategy after the first scan; Conservative: no major intracranial surgery throughout the entire clinical course.

Most participants were admitted to the ICU (355/461, 77%) (Table 2). Early targeted EDH evacuation was performed in 99 participants (21%), including 70/86 participants (81%) with large EDHs (Figure 2, **Supplemental Digital Content 3** [http://links.lww.com/NEU/E229], **Supplemental Digital Content 4** [http://links.lww.com/NEU/E230], **Supplemental Digital Content 5** [http://links.lww.com/NEU/E231]). Delayed targeted EDH evacuation was performed in 28 participants, including 8/16 participants with large EDHs not operated on initially. In these 28 participants, the median EDH volume was 23 cm^3^ (IQR 7-35) on the first scan and 44 cm^3^ (IQR 29-60) on the last follow-up scan before surgery. Any early hematoma evacuation was performed in 134 participants (29%). Conservative treatment throughout hospitalization was used in 286 participants (62%) (**Supplemental Digital Content 3**, http://links.lww.com/NEU/E229).

**TABLE 2.** Management and Outcome Characteristics of all Participants With EDHs and by Presence of Concomitant ASDHs and/or IPHs on the First Scan

Characteristic	Total (n = 461)	Findings on the first scan		*P* value^a^	Missing values (%)
		Isolated EDH (n = 133)	Nonisolated EDH (n = 328)		
Management					
Admission stratum (%)				<.001	0.0
Emergency room	1 (0.2)	0 (0.0)	1 (0.3)		
Ward	105 (22.8)	52 (39.1)	53 (16.2)		
Intensive Care Unit	355 (77.0)	81 (60.9)	274 (83.5)		
Early targeted EDH evacuation^b^ (%)	99 (21.5)	22 (16.5)	77 (23.5)	.13	0.0
Early EDH evacuation^c^ (%)	111 (24.1)	23 (17.3)	88 (26.8)	.04	0.0
EDH evacuation at any time point^d^ (%)	147 (31.9)	31 (23.3)	116 (35.4)	.02	0.0
Any early hematoma evacuation^e^ (%)	134 (29.1)	23 (17.3)	111 (33.8)	.001	0.0
Any hematoma evacuation at any time point^f^ (%)	175 (38.0)	31 (23.3)	144 (43.9)	<.001	0.0
Any cranial surgery at any time point^g^ (%)	218 (47.3)	39 (29.3)	179 (54.6)	<.001	0.0
Outcome					
In-hospital mortality (%)	32 (7.4)	3 (2.3)	29 (9.5)	.02	5.9
WOLSM^h^	27 (84.4)	3 (100.0)	24 (82.8)	1.000	0.0
Following participant's living will directives	6 (28.6)	1 (33.3)	5 (27.8)	1.000	22.2
ICU length of stay, median [IQR], d	6 [2, 14]	2 [1, 6]	8 [2, 16]	<.001	22.3
Alive at discharge (n = 402)	6 [2, 14]	3 [1, 5]	8 [2, 18]	<.001	24.6
Hospital length of stay, median [IQR], d	11 [5, 24]	6 [4, 11]	14 [7, 31]	<.001	2.8
Alive at discharge (n = 402)	11 [5, 25]	6 [4, 11]	15 [7, 34]	<.001	0.0
Home discharge (%)	213 (57.7)	97 (80.2)	116 (46.8)	<.001	20.0
6-month GOSE score^i^ (%)				<.001	15.6
1 = Death	41 (10.5)	3 (2.7)	38 (13.6)		
2 = Vegetative state/3 = lower severe disability	34 (8.7)	4 (3.6)	30 (10.8)		
4 = Upper severe disability	25 (6.4)	6 (5.5)	19 (6.8)		
5 = Lower moderate disability	61 (15.7)	13 (11.8)	48 (17.2)		
6 = Upper moderate disability	60 (15.4)	25 (22.7)	35 (12.5)		
7 = Lower good recovery	73 (18.8)	24 (21.8)	49 (17.6)		
8 = Upper good recovery	95 (24.4)	35 (31.8)	60 (21.5)		
6-month QoLIBRI score, median [IQR]	71 [54, 83]	73 [55, 92]	71 [54, 83]	.10	46.0

ASDH, acute subdural hematoma; CSF, cerebrospinal fluid; EDH, epidural hematoma; GOSE, Glasgow Outcome Scale–Extended; ICU, intensive care unit; IPH, intraparenchymal hemorrhage; QoLIBRI, Quality of Life after Brain Injury questionnaire; WOLSM, withdrawal of life-sustaining measures.

a *P* values derived from χ^2^ statistics for categorical variables and Mann-Whitney *U* tests for continuous variables (all non-normally distributed), comparing the isolated and nonisolated EDH subgroups. The *P* value assessed the compatibility with the null hypothesis of no differences between the 2 subgroups.

b Any intervention scheduled and conducted after the first scan in which EDH evacuation was the main surgical indication (potentially with simultaneous evacuation of adjacent ASDHs and/or IPHs).

c Any intervention scheduled and conducted after the first scan in which EDH evacuation was performed, regardless of whether EDH evacuation was the main surgical indication (targeted and nontargeted early EDH evacuation).

d Any intervention in which EDH evacuation was performed, regardless of whether EDH evacuation was the main surgical indication, including delayed interventions conducted after clinical/radiologic deterioration and secondary cranial interventions.

e Any intervention scheduled and conducted after the first scan in which any mass lesion (EDH and/or ASDH and/or IPH) was evacuated/decompressed, regardless of which hematoma constituted the main surgical indication.

f Any intervention in which any mass lesion (EDH and/or ASDH and/or IPH) was evacuated/decompressed, regardless of which hematoma constituted the main surgical indication, including delayed interventions conducted after clinical/radiologic deterioration and secondary cranial interventions.

g Any cranial surgery, including relatively minor interventions such as debridement, depressed skull fracture elevation, ventriculostomy for CSF drainage, CSF shunt, etc.

h Withdrawal of mechanical ventilation, vasoactive medication, continuous venovenous hemofiltration, intravenous fluids.

i GOSE scores were assessed by in-person/telephonic interviews or postal questionnaires, and as such, a clear distinction between GOSE 2 (vegetative state) and GOSE 3 (lower severe disability) was not always possible. As a result of this, these 2 categories were combined, giving a seven-point ordinal scale. When possible, missing values were imputed centrally from GOSE scores recorded at different time points (2 weeks to 1 year after injury), using a multistate model.

Six-month GOSE scores were available for 389 participants (84%), of which 289/389 (74%) had scores ≥5, and 41/389 (11%) died. In 27 of the 32 participants who died in hospital (84%), a decision to withdraw life-sustaining measures was made. Less than a third of decisions were made after explicit directives in the participant's living will (Table 2).

### Isolated vs Nonisolated Epidural Hematomas

Participants with isolated EDH were younger (median age 32 years vs 44 years) and presented with higher GCS scores (median 15, IQR 12-15) compared with participants with nonisolated EDH (median 11, IQR 5-14). Cisternal compression, midline shift (MLS), brain herniation, intraventricular hemorrhage, and traumatic axonal injury were more frequent in the nonisolated subgroup (Table 1, **Supplemental Digital Content 2**, http://links.lww.com/NEU/E228).

Participants with nonisolated EDH compared with isolated EDH more often received targeted EDH evacuation and any hematoma evacuation, both in the early phase (23% vs 17%, 34% vs 17%, respectively) and throughout hospitalization (30% vs 22%, 44% vs 23%, respectively, Figure 2). Participants with nonisolated EDH had longer hospital and ICU lengths of stay, higher in-hospital mortality, and lower 6-month GOSE scores (Table 2, **Supplemental Digital Content 6**, http://links.lww.com/NEU/E232).

Adherence to BTF guidelines to evacuate EDH ≥30 cm^3^ was high for both isolated (79% early, 89% during hospitalization) and nonisolated subgroups (82% early, 91% during hospitalization). The initial conservative management recommendation was followed in 94% and 91% of eligible participants in the isolated and nonisolated subgroups, respectively (**Supplemental Digital Content 4** [http://links.lww.com/NEU/E230], **Supplemental Digital Content 5** [http://links.lww.com/NEU/E231]).

### Association of Clinical, Imaging Characteristics, and Study Site with Acute Surgical Interventions

Larger EDH volume, below the 30 cm^3^ threshold (OR per 1 cm^3^ increase: 1.19 [95% CI 1.14-1.24]), smaller concomitant ASDH volume (OR per 1 cm^3^ increase: 0.93 [95% CI 0.88-0.97]), and MLS (OR 6.63 [95% CI 1.99-22.15]) were associated with early targeted EDH evacuation in multivariable analysis (**Supplemental Digital Content 7** [http://links.lww.com/NEU/E233], Table 3). These 3 variables were also associated with any early hematoma evacuation, with concomitant ASDH volume changing effect direction (**Supplemental Digital Content 8** [http://links.lww.com/NEU/E234], **Supplemental Digital Content 9** [http://links.lww.com/NEU/E235]). Full multivariable models had R^2^ of 73% and 65% for the 2 interventions, respectively, which increased to 80% and 73% when adding a random intercept for site. In sites enrolling >10 participants (303 participants), the MOR was 2.08 (95% CI 1.01-3.48) for early targeted EDH evacuation and 2.36 (95% CI 1.53-3.56) for any early hematoma evacuation.

**TABLE 3.** Association of Baseline Clinical and Imaging Characteristics With Early Targeted Epidural Hematoma Evacuation

Characteristic	Descriptive statistics^a^		Unit for regression	Univariable (n = 461)	Multivariable (n = 461)	
	No ETEE (n = 362)	ETEE (n = 99)		OR (95% CI)	OR (95% CI)	R^2b^
Age, median [IQR], y	40 [24, 57]	41 [26, 54]	Per 10 years increase	1.00 (0.90-1.10)	1.10 (0.82-1.34)	0.05
Baseline GCS score, median [IQR]	13 [7, 15]	11 [7, 14]	Per point increase, <9	1.13 (0.98-1.30)	1.18 (0.90-1.56)	
			Per point increase, ≥9	**0.84 (0.74-0.96)**	0.87 (0.68-1.12)	
Baseline one/both unreactive pupils (%)	46 (13.5)	13 (13.8)	Present	0.93 (0.48-1.78)	0.73 (0.14-3.88)	
Focal neurologic deficit (%)	33 (11.5)	13 (17.8)	Present	1.46 (0.78-2.73)	0.90 (0.26-3.12)	
Major extracranial injury (%)	178 (49.2)	37 (37.4)	Present	**0.62 (0.39-0.97)**	1.31 (0.55-3.10)	
EDH volume^c^, median [IQR], cm^3^	4 [1, 8]	48 [26, 82]	Per cm^3^ increase, <30 cm^3^	**1.20 (1.15-1.24)**	**1.19 (1.14-1.24)**	0.69
			per cm^3^ increase, ≥30 cm^3^	**1.02 (1.00-1.03)**	1.01 (0.99-1.03)	
Temporal EDH^d^ (%)	192 (53.0)	73 (73.7)	Present	**2.49 (1.52-4.08)**	2.26 (0.97-5.29)	
ASDH^e^ (%)	163 (45.0)	34 (34.3)	Present	0.95 (0.56-1.61)	0.89 (0.36-2.20)	0.71
ASDH volume^c^, median [IQR], cm^3^	0 [0, 5]	0 [0, 0]	Per cm^3^ increase	**0.97 (0.94-1.00)**	**0.93 (0.88-0.97)**	
IPH^e^ (%)	223 (61.6)	69 (69.7)	Present	1.58 (0.95-2.62)	1.48 (0.58-3.78)	
IPH volume^c^, median [IQR], cm^3^	1 [0, 9]	1 [0, 8]	Per cm^3^ increase	0.99 (0.98-1.01)	1.00 (0.98-1.02)	
TSAH (%)	259 (71.5)	74 (74.7)	Present	1.18 (0.71-1.96)	1.66 (0.62-4.46)	
Midline shift (%)	41 (11.3)	54 (54.5)	Present	**9.40 (5.62-15.70)**	**6.63 (1.99-22.15)**	0.73
Cisternal compression (%)	96 (26.5)	60 (60.6)	Present	**4.26 (2.67-6.80)**	1.19 (0.46-3.06)	

ASDH, acute subdural hematoma; EDH, epidural hematoma; ETEE, early targeted EDH evacuation; GCS, Glasgow Coma Scale; IPH, intraparenchymal hemorrhage; OR, odds ratio; TSAH, traumatic subarachnoid hemorrhage.

a Containing missing values for baseline GCS score, baseline pupils, and focal neurologic deficit, as reported in Table 1. The univariable and multivariable regression models used imputed values.

b Model Nagelkerke pseudo-R^2^s were calculated for models including the covariates on the corresponding rows and the rows above in the table. For example, the 0.69 pseudo-R^2^ is calculated for the model including age, GCS (piecewise), pupil reactivity, focal neurologic deficit, major extracranial injury, EDH volume (piecewise), and temporal EDH. As more covariates are added to a model, the proportion of explained “variation” of the outcome, in this case whether early targeted EDH evacuation occurred, increases.

c Volumes of individual lesions were estimated using the width × depth × length × 0.5 formula. When multiple lesions of a given type were present simultaneously, their volumes were added up.

d EDH extending into the temporal region (eg, temporal, temporoparietal, temporofrontal) compared with EDH without extension into temporal region.

e Binary indicator variables for the presence or absence of ASDH and IPH were included to adjust the respective continuous volume variables, which displayed spikes at zero, in both “univariable” and multivariable analysis.

Preinjury systemic disease (according to American Society of Anesthesiologists–Physical Status classification system) and admission stratum were considered as potential predictors. Neither had a significant association in multivariable analysis, neither significantly changed the association estimates of the other covariates in the full model, and the proportion of explained variance did not increase with the addition of either.

Bold entries represent statistically significant association estimates.

## DISCUSSION

In this large, prospective, contemporary, multicenter cohort of patients with traumatic EDHs, more than two-thirds harbored concomitant ASDHs and/or IPHs. Participants with isolated EDHs were younger, presented with less severe clinical findings, received less surgery, and had better outcomes than participants with nonisolated EDHs. The BTF guideline recommendation to surgically evacuate EDHs ≥30 cm^3^ was generally followed in both subgroups. Larger EDH volume, concomitant ASDH volume, and MLS were associated with early targeted EDH evacuation and any early hematoma evacuation.

### Isolated vs Nonisolated Epidural Hematomas

Patients with heterogeneous intracranial lesions, arguably the most clinically challenging, are paradoxically the least studied. Only limited recommendations exist for their surgical management in current lesion-specific guidelines.^14,31,32^ While frequent ASDH-IPH co-occurrence is recognized, with recommendations to consider ASDH and IPH guidelines together,^32^ no such indication is given for EDHs—potentially driven by the belief that EDHs mainly occur in isolation. In our EDH cohort, concomitant ASDHs and/or IPHs were present in most participants. The proportion of nonisolated EDHs was larger than previously reported in conservative (42%),^33^ surgical (8%-45%),^11-13,34-39^ and combined cohorts (6%-60%)^3,6,7,40-44^ and might be a consequence of our inclusive selection. Our liberal inclusion was designed to overcome limitations of lesion-specific TBI studies that restricted selection to patients harboring the lesion of interest in isolation or in combination with “minor”/“mild” concomitant intracranial lesions (rarely reporting specific criteria to define “minor”/“mild”).

Our findings suggest 2 distinct phenotypes of structural injury, isolated and nonisolated EDH, with the previously recognized deleterious effects of concomitant lesions on outcomes of patients with EDH^6,10,11,34-40^ being reconfirmed. The isolated EDH phenotype closely resembled the classically described EDH profile of a younger patient expected to have good recovery. Only 3 participants with isolated EDH died, all harboring concomitant TSAH, meaning Bricolo's decades-old expectation of zero mortality for EDH^13^ was only attained for isolated EDH without TSAH. Conversely, the nonisolated EDH phenotype, vastly predominant in our study, had a median age over a decade older compared with historical cohorts (and the observed isolated EDH phenotype) and an inverted ratio of traffic accidents to falls as injury cause.^7,12,13,35-38,43-47^ This high prevalence of concomitant lesions and corresponding deviation from the typical EDH presentation could be the result of past exclusion/underreporting of concomitant hematomas or the reflection of EDH as a changed disease in high-income countries.^15-20^ It is possible that EDHs described 20 to 30 years ago were mainly isolated, resulting from unmitigated skull fractures after traffic incidents. Road traffic safety measures and head-protection implementation (helmets, seatbelts, airbags) may have changed the biomechanics and subsequent pathobiology of these injuries, explaining the distinct EDH profile observed in this contemporary dataset.

### Predictors of Acute Surgical Interventions and Brain Trauma Foundation Guideline Adherence

Given the large proportion of heterogeneous hematomas in our cohort, studying surgical decision-making was not straightforward. Some interventions targeted a non-EDH lesion, multiple ones simultaneously, or included evacuation of an EDH which—if isolated—might have been treated conservatively. We attempted to clarify the complexity across surgical indications by defining 2 interventions of interest, to distinguish between “any early hematoma evacuation” and those specifically targeting an EDH.

EDH volume “explained” a considerable proportion of observed variation in both acute interventions. In multivariable analysis, incremental EDH volume increases were associated with early targeted EDH evacuation only below the 30 cm^3^ threshold. Because most EDH ≥30 cm^3^ were evacuated, additional EDH volumes above this threshold conferred marginal increases in intervention likelihood. These results confirm that, in CENTER-TBI participating hospitals, the BTF guideline recommendation to evacuate EDHs ≥30 cm^3^ has withstood the test of time.^48^ Recent case reports/small series have reported good outcomes for selected patients with isolated EDH ≥30 cm^3^ treated conservatively and proposed revisiting the cut-off.^49-51^ In our study, half of the 16 participants with EDHs ≥30 cm^3^ initially treated conservatively received delayed evacuation. Outcomes in those treated conservatively throughout hospitalization varied from death (participant with extremely poor prognosis and treatment-limiting decision) to complete recovery.

The BTF recommendation for nonoperative management was also followed, except for a few early surgeries, mostly targeting non-EDH lesions or EDHs extending into the temporal region. Concomitant ASDHs and/or IPHs were previously identified^35,52^ as a surgical indication in patients with EDH, postulating a decreased intracranial compliance, additional to their independent pathologic effects. Consequently, EDHs that may otherwise be well-tolerated require more aggressive management and have worse prognosis.^6,35,36,53^ In multivariable analyses, larger concomitant ASDH volume was associated with increased likelihood of any early hematoma evacuation and decreased likelihood of early targeted EDH evacuation. Temporal EDH location was also previously recognized as a potential surgical indication^33,41,52,54-56^ and had a strong, albeit nonsignificant, association with early targeted EDH evacuation in multivariable analysis.

Despite BTF recommendations for emergency surgery, comatose participants with anisocoria received highly varied acute management. This subgroup, along with the subset of participants for whom no specific BTF recommendations exist (ie, small EDHs and some degree of clinical impairment), might have driven the observed practice variation. Insufficient case-mix adjustment of site effects is possible, despite large R^2^s of fixed-effects models. The observed variation enables further comparative effectiveness studies, particularly to identify patient subgroups most likely to benefit from emerging minimally invasive techniques.^57,58^

### Limitations

CENTER-TBI did not explicitly capture potential lesion prioritizations during surgical decision-making. Moreover, the sample size might be insufficient to definitively assess volume thresholds for surgery in patients with heterogeneous hematomas. We do not claim definitive answers regarding conditional surgical decision-making, let alone their impact on patient outcomes, which was beyond the scope of this work. Without further analysis of outcomes, no conclusions can be drawn based on this work on the effectiveness of acute interventions or the BTF guideline recommendations. Modeling surgical decision-making beyond the acute phase was limited by the lack of detailed information on follow-up scans. Our results, derived from a relatively homogenous White European patient population, treated in neurotrauma referral centers, might have limited generalizability. Treatment-limiting decisions in patients with extremely poor prognosis and patient, family, and/or caregiver management preferences were neither excluded from the analyses nor separately analyzed. Our EDH cohort represents a convenience sample within CENTER-TBI, without prior EDH-specific power calculations. Nonetheless, this study represents one of the largest reported EDH cohorts to date.

## CONCLUSION

In current practice, isolated EDHs are relatively infrequent, with two-thirds of patients presenting with concomitant ASDHs and/or IPHs. Restrictive lesion/patient selection might create a simplistic view of real-world practice and perpetuate blind spots in TBI knowledge. Isolated EDHs (and potentially isolated ASDHs and IPHs too) might have become the exception and not the rule, meaning that lesion-specific guidelines may be less applicable to current practice. Based on our findings, on factors influencing early decision-making, future research should examine the effectiveness of acute surgery and BTF guideline adherence on the outcomes of patients with EDH, isolated or not. Alternatively, future research could deploy a holistic approach, moving past the current analytical framework of categorizing patients with TBI into (partially overlapping) “EDH,” “ASDH,” “IPH” subgroups. Confounding bias when studying decision-making and treatment effectiveness should be minimized by including, reporting, and quantitatively modeling coexisting intracranial injuries, regardless of their size or apparent clinical significance. We recommend such complex, holistic future research in patients with heterogeneous TBI lesions, beyond the EDH population focused on here. The clustering and interplay among different types of injuries, present in varying quantities, and with independent, potentially nonlinear effects on pathologic intracranial lesion burden, surgical indication, and outcome, should be investigated and quantified. Only then can guidelines be updated to account for the complexity of managing patients with heterogeneous hematomas.

## Supplementary Material

**Figure:**
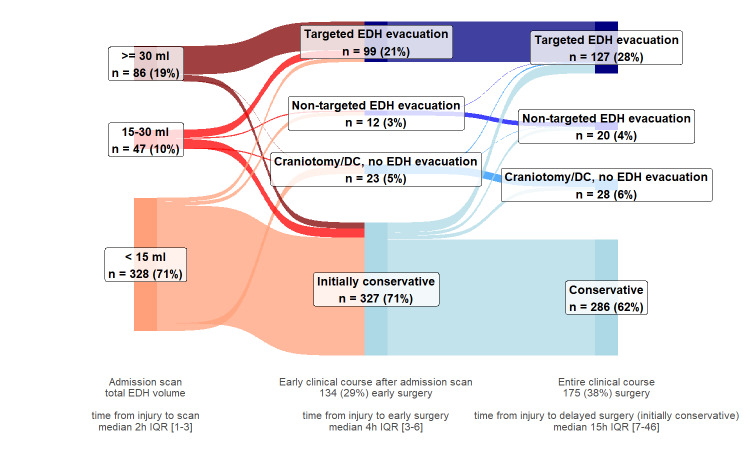


**Figure:**
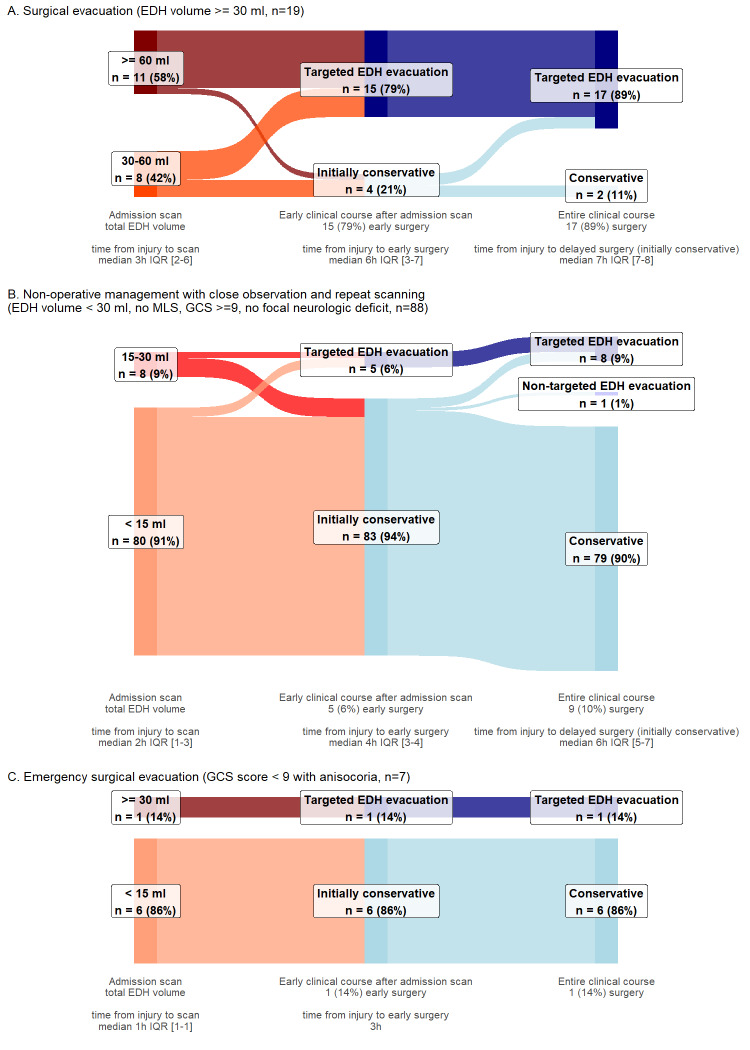


**Figure:**
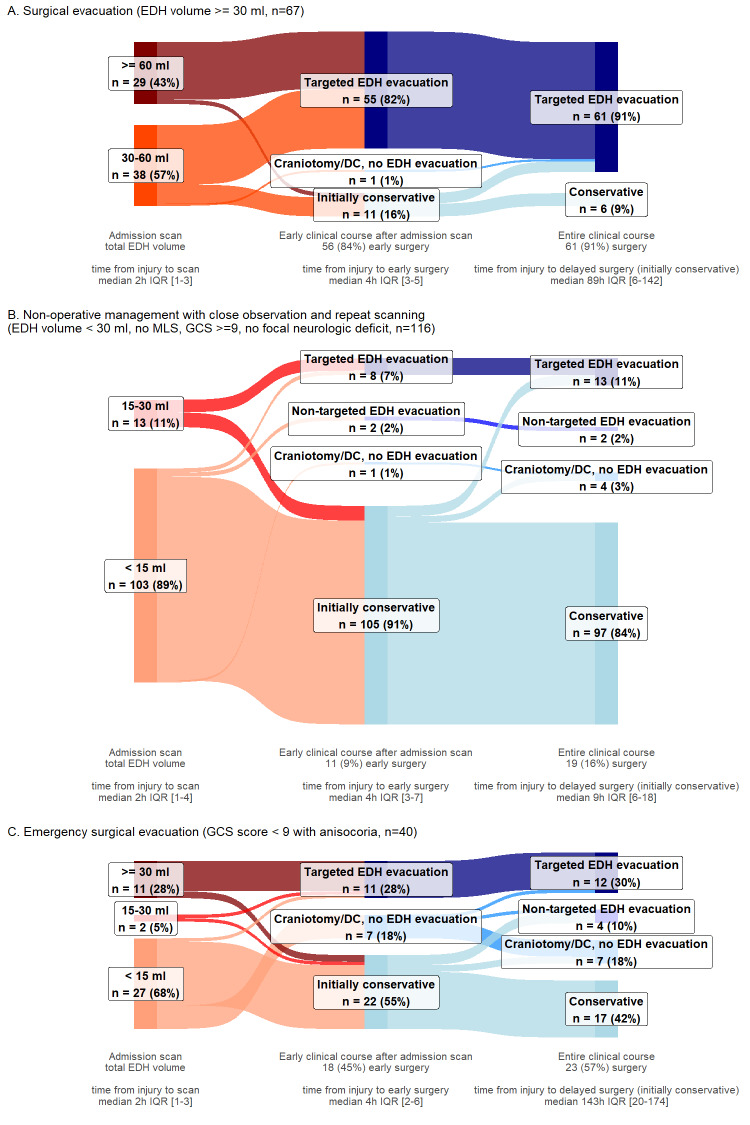


**Figure:**
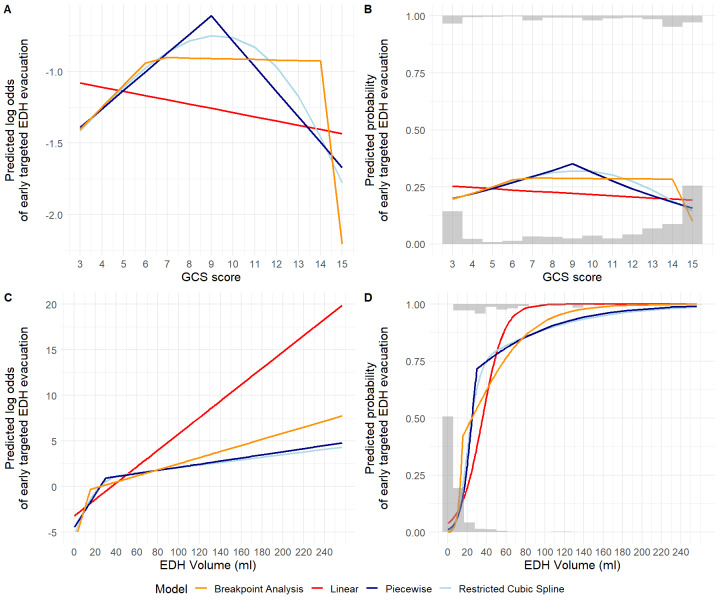


**Figure:**
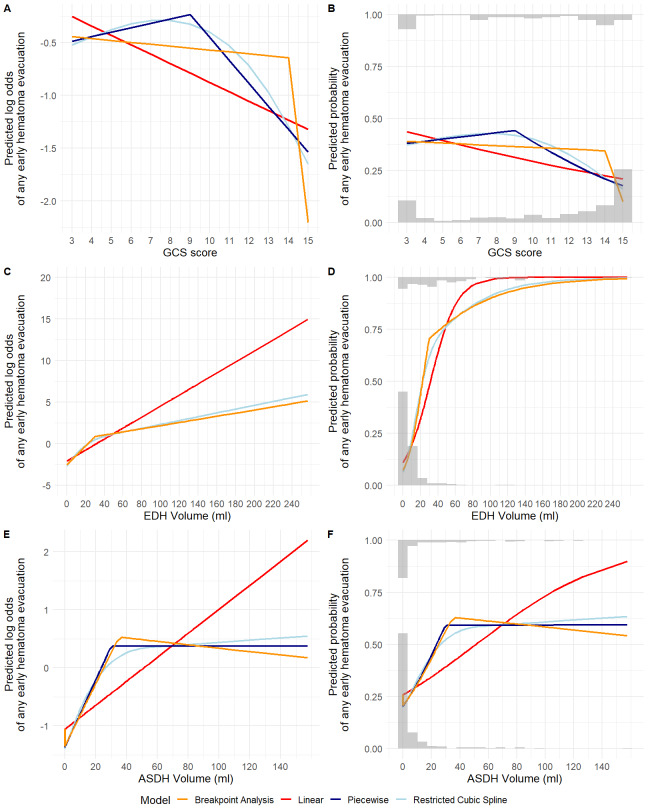


## SUPPLEMENTAL DIGITAL CONTENT

**Supplemental Digital Content 1**. **Supplemental Methods**. Additional details about ethical approval for CENTER-TBI, the interventions, outcomes, Sankey diagram interpretation, and regression modeling. **Supplemental Digital Content 2**. **Table**. Additional Baseline Clinical and Imaging Characteristics of all Participants with Epidural Hematomas and by Presence of Concomitant Acute Subdural Hematomas and/or Intraparenchymal Hemorrhages on the First Scan. **Supplemental Digital Content 3**. **Figure**. Surgical Care Pathways of Participants with Epidural Hematomas (n = 461). Most EDHs in our sample had total volumes <15 cm^3^ (328 participants, 71%), most of which were treated conservatively throughout the entire clinical course (265 participants, 81%). Early targeted EDH evacuation was mostly performed for EDHs ≥30 cm^3^ (70/99 participants, 71%) but also in participants with smaller lesions, including 10 participants with EDHs <15 cm^3^. An initial conservative strategy, followed by delayed surgery was used in 41 participants (9%), most of whom had small EDHs (61% had EDHs <15 cm^3^). Delayed targeted EDH evacuation was performed in 28 participants, with a median EDH volume of 23 cm^3^ (IQR 7-35) on the first scan and 44 cm^3^ (IQR 29-60) on the last follow-up scan before targeted EDH evacuation. Abbreviations and definitions: DC, decompressive craniectomy; EDH, epidural hematoma; IQR, interquartile range. Targeted EDH evacuation: EDH evacuation was the main surgical indication; Nontargeted EDH evacuation: EDH evacuation performed, but was not the main surgical indication; Craniotomy/DC, no EDH evacuation: craniotomy or DC for ASDH and/or IPH evacuation/decompression, during which the EDH was not evacuated; Initially conservative: initial nonsurgical management strategy after the first scan; Conservative: no major intracranial surgery throughout the entire clinical course. **Supplemental Digital Content 4**. **Figure**. Surgical Care Pathways of Participants with Isolated Epidural Hematoma, by corresponding Brain Trauma Foundation Guideline Recommendation. A. Surgical evacuation (EDH volume ≥30 cm^3^, n = 19): Most isolated EDHs ≥30 cm^3^ were treated by early targeted EDH evacuation. Two participants were not operated at any timepoint because of acceptable/good neurologic condition (one with complete recovery at 6 months and one lost to follow-up). In 2 other participants, the evacuation was delayed, either by the placement of an intraparenchymal pressure monitor or by the surgeon being delayed. In both participants, a repeat scan before the delayed surgery revealed EDH enlargement. All isolated EDHs ≥60 cm^3^ were evacuated, if not early, then later in the clinical course. B. Nonoperative management with close observation and repeat scanning (EDH volume <30 cm^3^, no MLS, GCS ≥9, no focal neurologic deficit, n = 88): Of 88 participants with isolated EDH fulfilling at baseline the Brain Trauma Foundation guideline criteria for nonoperative management, 83 (94%) received initial conservative treatment. Five participants received early targeted EDH evacuation, motivated by clinical deterioration, mass effect on CT, or guideline adherence. In all 5, the EDH was extending into the temporal region. Three participants initially treated conservatively required delayed targeted EDH evacuation, with EDH volumes ranging from 10 to 26 cm^3^ on the first scan, and from 24 to 90 cm^3^ on the last follow-up scan before surgery. C. Emergency surgical evacuation (GCS score <9 with anisocoria, n = 7): Seven participants with isolated EDH presented in a coma with anisocoria. One participant with a large EDH (184 cm^3^) received emergency/life-saving early targeted EDH evacuation. The rest had EDHs <15 cm^3^ and did not receive any acute surgery, motivated by little/no mass effect, or no surgical lesions on the first CT. Abbreviations and definitions: EDH, epidural hematoma; GCS, Glasgow Coma Scale; IQR, interquartile range; MLS, midline shift. Targeted EDH evacuation: EDH evacuation was the main surgical indication; Nontargeted EDH evacuation: EDH evacuation performed, but was not the main surgical indication; Initially conservative: initial nonsurgical management strategy after the first scan; Conservative: no major intracranial surgery throughout the entire clinical course. **Supplemental Digital Content 5**. **Figure**. Surgical Care Pathways of Participants with Nonisolated Epidural Hematoma, by corresponding Brain Trauma Foundation Guideline Recommendation. A. Surgical evacuation (EDH volume ≥30 cm^3^, n = 67): Most nonisolated EDHs ≥30 cm^3^ were treated by early targeted EDH evacuation. Six participants were not operated at any timepoint, including one participant with a 128 cm^3^ EDH considered to have extremely poor prognosis after the first scan and who later died during hospitalization. In the other 5 participants treated conservatively, with EDH volumes ranging from 32 to 51 cm^3^, no surgery was indicated after the first scan, motivated by acceptable/good neurologic condition, absence of a surgical lesion, or little/no mass effect. Their 6-month GOSE scores varied from 2/3 to 8. Delayed targeted EDH evacuation was performed in 6 participants, including one participant who first received a craniotomy for contralateral ASDH. Nonisolated EDHs ≥60 cm^3^ were almost always evacuated, if not early (93%), then later in the clinical course (97%). B. Nonoperative management with close observation and repeat scanning (EDH volume <30 cm^3^, no MLS, GCS ≥9, no focal neurologic deficit, n = 116): Of 116 participants with nonisolated EDH fulfilling at baseline the Brain Trauma Foundation guideline criteria for nonoperative management, 105 (91%) received initial conservative treatment. Eight participants received early targeted EDH evacuation, motivated by mass effect on CT or clinical deterioration. In 6 of these, the EDH was extending into the temporal region. Five participants initially treated conservatively required delayed targeted EDH evacuation. C. Emergency surgical evacuation (GCS score <9 with anisocoria, n = 40): Forty participants with nonisolated EDH presented in a coma with anisocoria. Emergency surgery was performed in 18 of them (45%), either for targeted EDH evacuation (median EDH volume on first scan 49 cm^3^, IQR 34-74) or for ASDH evacuation by craniotomy/DC, without EDH evacuation (range ASDH volume on first scan 20-126 cm^3^). Seventeen participants (42%) were treated conservatively throughout the entire clinical course, including 5 participants with extremely poor prognosis as judged by the treating physician based on the first scan. In the other 12 participants, the decision to treat conservatively was motivated by acceptable/good neurologic condition, absence of surgical lesion, or little/no mass effect on the first scan (median EDH, ASDH, IPH sum volume on first scan 20 cm^3^, IQR 5-33). Abbreviations and definitions: DC, decompressive craniectomy; EDH, epidural hematoma; GCS, Glasgow Coma Scale; IQR, interquartile range; MLS, midline shift. Targeted EDH evacuation: EDH evacuation was the main surgical indication; Non-targeted EDH evacuation: EDH evacuation performed, but was not the main surgical indication; Craniotomy/DC, no EDH evacuation: craniotomy or DC for ASDH and/or IPH evacuation/decompression, during which the EDH was not evacuated; Initially conservative: initial nonsurgical management strategy after the first scan; Conservative: no major intracranial surgery throughout the entire clinical course. **Supplemental Digital Content 6**. **Table**. Additional Management and Outcome Characteristics of all Participants with Epidural Hematomas and by Presence of Concomitant Acute Subdural Hematomas and/or Intraparenchymal Hemorrhages on the First Scan. **Supplemental Digital Content 7**. **Figure**. Nonlinear Relationships of GCS Score and EDH Volume with Early Targeted Epidural Hematoma Evacuation. Of all continuous independent variables considered, GCS score and EDH volume demonstrated significantly nonlinear univariable relationships with log odds of early targeted EDH evacuation. For each variable, linear (red regression line) and restricted cubic spline (light blue regression line) univariable models were constructed and compared with the likelihood ratio test (*P* values of 0.003 and <0.001 for GCS score and EDH volume, respectively). Linear relationships in the log odds space (panels A, C) translate to smooth lines in the probabilities space (panels B, D). Furthermore, in a data-driven approach, we conducted breakpoint analysis to identify the number of and exact values where changes of effect occur (yellow regression line). For GCS score, 2 breakpoints were identified, at approximately 6 and 14 points. These can be interpreted as a slight increase in intervention probability from GCS score 3 to 6 (OR 1.18, 95% CI 0.89-1.56 per point increase), followed by a relatively constant probability for GCS values between 6 and 14 (OR 1.00, 95% CI 0.90-1.11 per point increase) and a decreased probability for GCS 15 (OR 0.28, 95% CI 0.13-0.55 for transitions from GCS score 14–15). In multivariable analysis, adjusting for the other candidate predictors of early targeted EDH evacuation, GCS score modeled using the data-derived breakpoints was not associated with the intervention. This is consistent with the relatively modest overall association of GCS score with the intervention (panel B: predicted probabilities of the intervention for the entire GCS score range are close to the observed mean: 0.21). For EDH volume, 2 breakpoints were detected, at approximately 3 and 15 cm^3^. The probability of early targeted EDH evacuation was estimated to be zero below 3 cm^3^, increased steeply up to 15 cm^3^ (OR 1.46, 95% CI 1.29-1.72 per cm^3^ increase), followed by a decelerated increase above 15 cm^3^ (OR 1.03, 95% CI 1.02-1.03 per cm^3^ increase). In multivariable analysis, adjusting for the other candidate predictors of early targeted EDH evacuation, EDH volume modeled using the data-derived breakpoints was associated with the intervention, with similar piecewise association magnitudes as in univariable analysis. To approximate the nonlinear nature of these relationships in subsequent modeling, we used piecewise regression (dark blue regression line). In piecewise regression, a threshold value is used to split the range of a continuous predictor, thus allowing for different statistical effect estimates to be computed for incremental increases of the continuous variable, below and above the threshold. These thresholds were set according to clinical significance at 9 points for GCS (cut-off between comatose vs noncomatose status) and 30 cm^3^ for EDH volume (volume cut-off for surgical indication according to the Brain Trauma Foundation guideline). Abbreviations: EDH, epidural hematoma; GCS, Glasgow Coma Scale. **Supplemental Digital Content 8**. **Figure**. Nonlinear Relationships of GCS Score, EDH Volume, and ASDH volume with Any Early Hematoma Evacuation. Of all continuous independent variables considered, GCS score, EDH volume, and ASDH volume demonstrated significantly nonlinear univariable relationships with log odds of any early hematoma evacuation. For each variable, linear (red regression line) and restricted cubic spline (light blue regression line) models were constructed and compared with the likelihood ratio test (*P* values of 0.004, <0.001, and 0.03 for GCS score, EDH volume, and ASDH volume, respectively). The compared models for GCS score and EDH volume were univariable, while the models for ASDH volume contained an additional binary variable for presence of ASDH, to adjust the continuous volume variable, which displayed spike at zero. Linear relationships in the log odds space (panels A, C, E) translate to smooth lines in the probabilities space (panels B, D, F). Furthermore, in a data-driven approach, we conducted breakpoint analysis to identify the number of and exact values where changes of effect occur (yellow regression line). For GCS score, a single breakpoint was identified, at approximately 14 points. This can be interpreted as a slight decrease in intervention probability from GCS score 3 to 14 (OR 0.98, 95% CI 0.93-1.04 per point increase), followed by a steeply decreased probability for GCS 15 (OR 0.21, 95% CI 0.10-0.40 for transitions from GCS score 14 to 15). In multivariable analysis, adjusting for the other candidate predictors of any early hematoma evacuation, the associations of GCS score modeled using the data-derived breakpoint remained consistent with the ones from univariable analysis. For EDH volume, a single breakpoint was identified, at approximately 30 cm^3^, coinciding with the clinically significant threshold that was selected for piecewise regression in subsequent modeling (results in **Supplemental Digital Content 9** [http://links.lww.com/NEU/E235]). For ASDH volume, a single breakpoint was identified, at approximately 35 cm^3^. Adjusted for presence of ASDH, the probability of any early hematoma evacuation increased from ASDH volume 0 to 35 cm^3^ (OR 1.06, 95% CI 1.03-1.09 per cm^3^ increase), followed by a plateau above 35 cm^3^ (OR 1.00, 95% CI 0.98-1.02 per cm^3^ increase). In multivariable analysis, adjusting for the other candidate predictors of any early hematoma evacuation, the associations of ASDH volume modeled using the data-derived breakpoint remained consistent with the ones from univariable analysis. To approximate the nonlinear nature of these relationships in subsequent modeling, we used piecewise regression (dark blue regression line). In piecewise regression, a threshold value is used to split the range of a continuous predictor, thus allowing for different statistical effect estimates to be computed for incremental increases of the continuous variable, below and above the threshold. These thresholds were set according to clinical significance at 9 points for GCS (cut-off between comatose vs noncomatose status), 30 cm^3^ for EDH volume (volume cut-off for surgical indication according to the Brain Trauma Foundation guideline, coinciding with the data-derived breakpoint), and 30 cm^3^ for ASDH volume. Abbreviations: ASDH, acute subdural hematoma; EDH, epidural hematoma; GCS, Glasgow Coma Scale. **Supplemental Digital Content 9**. **Table**. Association of Baseline Clinical and Imaging Characteristics with Any Early Hematoma Evacuation.
