# Supplementary material for: Clinical and Imaging Characteristics, Care Pathways, and Outcomes of Traumatic Epidural Hematomas: A Collaborative European NeuroTrauma Effectiveness Research in Traumatic Brain Injury Study
Source: Neurosurgery. 2024 May 21;95(5):986–99. doi: 10.1227/neu.0000000000002982 (PMC11449426; doi:10.1227/neu.0000000000002982)
Supplement: Supplementary file 1 [file neu-95-0986-s001.docx]

**Supplemental Digital Content 1, Methods.**

The CENTER-TBI study (EC grant 602150) has been conducted in accordance with all relevant laws of the EU if directly applicable or of direct effect and all relevant laws of the country where the Recruiting sites were located, including but not limited to, the relevant privacy and data protection laws and regulations (the “Privacy Law”), the relevant laws and regulations on the use of human materials, and all relevant guidance relating to clinical studies from time to time in force including, but not limited to, the ICH Harmonised Tripartite Guideline for Good Clinical Practice (CPMP/ICH/135/95) (“ICH GCP”) and the World Medical Association Declaration of Helsinki entitled “Ethical Principles for Medical Research Involving Human Subjects”. Informed Consent by the patients and/or the legal representative/next of kin was obtained, accordingly to the local legislations, for all patients recruited in the Core Dataset of CENTER-TBI and documented in the e-CRF. Ethical approval was obtained for each recruiting site. The list of sites, Ethical Committees, approval numbers and approval dates can be found on the website: https://www.center-tbi.eu/project/ethical-approval

Surgical intervention groups were defined by surgical indication after the first scan, surgery start time, code, description and notes, and visual confirmation by comparing pre- and post-operative scans. Surgeries for hematoma evacuation and/or mass effect consisted of craniotomies and craniectomies.

The Glasgow Outcome Scale-Extended assesses global functional recovery after TBI and is widely used as the primary outcome measure in TBI studies.^1,2^ Participants are assigned an ordinal score from 1 to 8: 1=death, 2=vegetative state, 3=lower severe disability, 4=upper severe disability, 5=lower moderate disability, 6=upper moderate disability, 7=lower good recovery, 8=upper good recovery.

Sankey diagrams were created to visualize surgical management pathways, depicting participant trajectories across 3 stages: (1) total epidural hematoma (EDH) volume on first scan (3 categories: < 15 cm^3^, 15-30 cm^3^, ≥ 30 cm^3^), (2) early clinical course after first scan, and (3) entire clinical course, including delayed/follow-up interventions.^3^ In the diagrams, the absolute and relative frequencies in the categories of each stage were reported. Transitions between stages were depicted as flows, proportional to the corresponding numbers of participants. Diagrams were also created for participant subgroups defined according to the Brain Trauma Foundation guideline recommendation categories: a) surgical evacuation (participants with EDH volume ≥ 30 cm^3^), b) non-operative management with close observation and repeat scanning (participants with EDH volume < 30 cm^3^, no midline shift, Glasgow Coma Score (GCS) score ≥ 9, no focal neurologic deficit) and c) emergency surgical evacuation (participants with GCS score < 9 with anisocoria).

To test the linearity assumption of the relationships between continuous independent variables and log odds of the interventions, linear and restricted cubic splines univariable models were constructed and compared with the likelihood ratio test. Variables with significantly non-linear relationships were coded piecewise in subsequent modeling. In piecewise regression, a threshold value is selected to split the range of a continuous independent variable, thus allowing for different statistical effect estimates to be computed for incremental increases of the continuous variable, below and above the threshold. Thresholds were set according to clinical significance. Furthermore, in a data-driven approach, we conducted breakpoint analysis to identify the number of and exact values along the range of the continuous variables where changes of statistical effect occur, and further evaluated these different association estimates in multivariable analysis.

To assess the additional “variation” in the two interventions that could be explained by study site, the full fixed-effects models in the entire sample were further extended with site as a random intercept and the Nagelkerke pseudo-R^2^s of these extended models were reported.

The median odds ratio (MOR) is a summary measure of practice variation and is defined as the median of the set of odds ratios that could be obtained by comparing two identical participants from two randomly chosen sites. A MOR=1 indicates no variation in site policies. For sites enrolling > 10 participants, the MOR was derived from mixed-effects logistic regression with a random intercept for site and adjustment for baseline variables with p<0.2 in the full fixed-effects model. MOR 95% CIs were derived from profile likelihood CIs of the random effect standard deviations.

**REFERENCES**

1. Wilson JT, Pettigrew LE, Teasdale GM. Structured interviews for the Glasgow Outcome Scale and the extended Glasgow Outcome Scale: guidelines for their use. *J Neurotrauma*. Aug 1998;15(8):573-85.

2. Wilson L, Boase K, Nelson LD, et al. A Manual for the Glasgow Outcome Scale-Extended Interview. *J Neurotrauma*. Sep 1 2021;38(17):2435-2446.

3. *ggsankey: Sankey, Alluvial and Sankey Bump Plots*. Version R package 0.0.99999. David Sjoberg; 2022.
