# Supplementary material for: Clinical and Imaging Characteristics, Care Pathways, and Outcomes of Traumatic Epidural Hematomas: A Collaborative European NeuroTrauma Effectiveness Research in Traumatic Brain Injury Study
Source: Neurosurgery. 2024 May 21;95(5):986–99. doi: 10.1227/neu.0000000000002982 (PMC11449426; doi:10.1227/neu.0000000000002982)
Supplement: Supplementary file 2 [file neu-95-0986-s002.docx]

**Supplemental Digital Content 2, Table. Additional Baseline Clinical and Imaging Characteristics of all Participants with Epidural Hematomas and by Presence of Concomitant Acute Subdural Hematomas and/or Intraparenchymal Hemorrhages on the First Scan**

| Characteristic | Total (n=461) | Findings on the first scan | | P value^a^ | Missing values (%) |
| --- | --- | --- | --- | --- | --- |
|  |  | Isolated EDH (n=133) | Non-isolated EDH (n=328) |  |  |
| **Clinical** | | | | | |
| Pre-injury systemic disease, ASA-PS classification^b^ (%) | 139 (31.3) | 32 (24.4) | 107 (34.2) | 0.06 | 3.7 |
| Anticoagulants and/or platelet aggregation inhibitors (%) | 28 (6.4) | 5 (3.8) | 23 (7.5) | 0.22 | 4.6 |
| Injury type (%) |  |  |  | 0.38 | 1.3 |
| Closed | 406 (89.2) | 118 (88.7) | 288 (89.4) |  |  |
| Crush | 14 (3.1) | 2 (1.5) | 12 (3.7) |  |  |
| Penetrating | 11 (2.4) | 5 (3.8) | 6 (1.9) |  |  |
| Closed with open depressed skull fracture | 24 (5.3) | 8 (6.0) | 16 (5.0) |  |  |
| Loss of consciousness (%) |  |  |  | 0.04 | 14.1 |
| Yes | 217 (54.8) | 55 (48.2) | 162 (57.4) |  |  |
| Suspected | 52 (13.1) | 12 (10.5) | 40 (14.2) |  |  |
| Seizures (%) | 33 (7.5) | 8 (6.2) | 25 (8.0) | 0.65 | 4.1 |
| Vomiting (%) | 123 (30.2) | 37 (30.1) | 86 (30.3) | 1.00 | 11.7 |
| Brain injury AIS, median[IQR] | 4 [3, 5] | 4 [3, 4] | 5 [4, 5] | <0.001 | 1.5 |
| ISS, median [IQR] | 25 [16, 38] | 21 [16, 32] | 26 [16, 41] | <0.001 | 1.5 |
| **Imaging** | | | | | |
| Likely bleeding source^c^ (%) |  |  |  | 0.28 | 5.6 |
| Arterial | 279 (64.1) | 85 (67.5) | 194 (62.8) |  |  |
| Venous | 113 (26.0) | 33 (26.2) | 80 (25.9) |  |  |
| Mixed sources | 43 (9.9) | 8 (6.3) | 35 (11.3) |  |  |
| EDH in parietal region (%) | 160 (34.7) | 41 (30.8) | 119 (36.3) | 0.31 | 0.0 |
| EDH in occipital region (%) | 41 (8.9) | 12 (9.0) | 29 (8.8) | 1.00 | 0.0 |
| EDH in posterior fossa (%) | 34 (7.4) | 9 (6.8) | 25 (7.6) | 0.90 | 0.0 |
| ASDH volume^d^, median (IQR), cm^3^ | 0 [0, 4] | NA | 0 [0, 8] | NA | 0.0 |
| IPH volume^e^, median (IQR), cm^3^ | 1 [0, 9] | NA | 4 [1, 15] | NA | 0.0 |
| Total EDH, ASDH, IPH volume^f^, median (IQR), cm^3^ | 19 [5, 50] | 4 [2, 13] | 29 [10, 59] | <0.001 | 0.0 |
| Intraventricular hemorrhage (%) | 74 (16.1) | 8 (6.0) | 66 (20.1) | <0.001 | 0.0 |
| Brain herniation (%) | 88 (19.1) | 14 (10.5) | 74 (22.6) | 0.004 | 0.0 |
| Diffuse/Traumatic axonal injury (%) | 38 (8.2) | 2 (1.5) | 36 (11.0) | 0.002 | 0.0 |
| Ventricular compression (%) | 146 (31.7) | 24 (18.0) | 122 (37.2) | <0.001 | 0.0 |
| Cortical sulcus effacement (%) | 98 (21.3) | 9 (6.8) | 89 (27.1) | <0.001 | 0.0 |
| *Abbreviations: AIS, Abbreviated Injury Scale (range 1-6, 1 indicating minor injury and 6 indicating fatal injury); ASA-PS, American Society of Anesthesiologists - Physical Status classification system; ASDH, acute subdural hematoma; EDH, epidural hematoma; IPH, intraparenchymal hemorrhage; IQR, interquartile range; ISS, Injury Severity Score (range 1-75, 1 indicating minimum injury severity and 75 indicating maximum injury severity); NA, not applicable.*  ^a^P values derived from χ² statistics for categorical variables and Mann-Whitney U tests for continuous variables (all non-normally distributed), comparing the isolated and non-isolated EDH subgroups. The p value assessed the compatibility with the null hypothesis of no differences between the two subgroups.  ^b^Mild systemic disease, severe systemic disease or severe systemic disease that is a constant threat to life.  ^c^CT can only approximate the bleeding source.  ^d^Volumes of individual lesions were estimated using the width × depth × length × 0.5 formula. When multiple ASDHs were present simultaneously, their volumes were added up.  ^e^Volumes of individual lesions were estimated using the width × depth × length × 0.5 formula. When multiple IPHs were present simultaneously, their volumes were added up.  ^f^Sum of total EDH, ASDH and IPH volumes. | | | | | |
