# Supplementary material for: Clinical and Imaging Characteristics, Care Pathways, and Outcomes of Traumatic Epidural Hematomas: A Collaborative European NeuroTrauma Effectiveness Research in Traumatic Brain Injury Study
Source: Neurosurgery. 2024 May 21;95(5):986–99. doi: 10.1227/neu.0000000000002982 (PMC11449426; doi:10.1227/neu.0000000000002982)
Supplement: Supplementary file 3 [file neu-95-0986-s006.docx]

**Supplemental Digital Content 6, Table. Additional Management and Outcome Characteristics of all Participants with Epidural Hematomas and by Presence of Concomitant Acute Subdural Hematomas and/or Intraparenchymal Hemorrhages on the First Scan**

| Characteristic | Total (n=461) | Findings on the first scan | | P value^a^ | Missing values (%) |
| --- | --- | --- | --- | --- | --- |
|  |  | Isolated EDH (n=133) | Non-isolated EDH (n=328) |  |  |
| **Management** | | | | | |
| ICP Monitor (%) | 177 (38.5) | 19 (14.3) | 158 (48.3) | <0.001 | 0.2 |
| Sustained raised ICP^b^ (%) |  |  |  | 0.36 | 62.0 |
| No | 85 (48.6) | 12 (63.2) | 73 (46.8) |  |  |
| Yes, controlled | 70 (40.0) | 6 (31.6) | 64 (41.0) |  |  |
| Yes, refractory | 20 (11.4) | 1 (5.3) | 19 (12.2) |  |  |
| Any raised ICP^c^ (%) | 113 (27.2) | 7 (5.7) | 106 (36.2) | <0.001 | 9.8 |
| Median daily TIL, median [IQR] | 2 [0, 6] | 1 [0, 2] | 3 [0, 8] | <0.001 | 23.4 |
| Maximum daily TIL, median [IQR] | 5 [1, 11] | 1 [0, 5] | 6 [2, 12] | <0.001 | 23.4 |
| Extra-cranial surgery (%) | 99 (21.7) | 26 (19.7) | 73 (22.5) | 0.59 | 1.1 |
| **Outcome** | | | | | |
| Any neuroworsening episode^d^ (%) | 111 (27.1) | 16 (12.8) | 95 (33.3) | <0.001 | 11.1 |
| Any lesion progression on CT^d^ (%) | 104 (26.1) | 13 (10.7) | 91 (32.9) | <0.001 | 13.4 |
| Increase in initial lesion | 81 (77.9) | 8 (61.5) | 73 (80.2) | 0.25 | 77.4 |
| Development of new lesion | 26 (25.0) | 5 (38.5) | 21 (23.1) | 0.39 | 77.4 |
| Any respiratory systemic complication^d^ (%) | 81 (20.8) | 6 (4.8) | 75 (28.3) | <0.001 | 15.6 |
| Ventilator associated pneumonia^e^ (%) | 47 (13.1) | 1 (1.2) | 46 (16.8) | <0.001 | 22.1 |
| Cause of death^f^ (%) |  |  |  | 0.65 | 92.2 |
| Head injury/initial injury | 23 (63.9) | 2 (66.7) | 21 (63.6) |  |  |
| Head injury/secondary intracranial damage | 6 (16.7) | 0 (0.0) | 6 (18.2) |  |  |
| Systemic trauma/Medical complications/Other | 7 (19.4) | 1 (33.3) | 6 (18.2) |  |  |
| 3-month GOSE score^g^ (%) |  |  |  | <0.001 | 16.1 |
| 1 = death | 37 (9.6) | 3 (2.7) | 34 (12.3) |  |  |
| 2 = vegetative state/3 = lower severe disability | 48 (12.4) | 5 (4.5) | 43 (15.5) |  |  |
| 4 = upper severe disability | 51 (13.2) | 14 (12.7) | 37 (13.4) |  |  |
| 5 = lower moderate disability | 47 (12.1) | 9 (8.2) | 38 (13.7) |  |  |
| 6 = upper moderate disability | 69 (17.8) | 24 (21.8) | 45 (16.2) |  |  |
| 7 = lower good recovery | 49 (12.7) | 20 (18.2) | 29 (10.5) |  |  |
| 8 = upper good recovery | 86 (22.2) | 35 (31.8) | 51 (18.4) |  |  |
| 12-month GOSE score^g^ (%) |  |  |  | <0.001 | 15.6 |
| 1 = death | 43 (11.1) | 3 (2.7) | 40 (14.3) |  |  |
| 2 = vegetative state/3 = lower severe disability | 31 (8.0) | 2 (1.8) | 29 (10.4) |  |  |
| 4 = upper severe disability | 19 (4.9) | 5 (4.5) | 14 (5.0) |  |  |
| 5 = lower moderate disability | 33 (8.5) | 5 (4.5) | 28 (10.0) |  |  |
| 6 = upper moderate disability | 70 (18.0) | 26 (23.6) | 44 (15.8) |  |  |
| 7 = lower good recovery | 72 (18.5) | 22 (20.0) | 50 (17.9) |  |  |
| 8 = upper good recovery | 121 (31.1) | 47 (42.7) | 74 (26.5) |  |  |
| *Abbreviations: EDH, epidural hematoma; GOSE, Glasgow Outcome Scale – Extended; ICP, intracranial pressure; IQR, interquartile range; TIL, Therapy Intensity Level (for ICP management).*  ^a^P values derived from χ² statistics for categorical variables and Mann-Whitney U tests for continuous variables (all non-normally distributed), comparing the isolated and non-isolated EDH subgroups. The p value assessed the compatibility with the null hypothesis of no differences between the two subgroups.  ^b^In participants with brain specific monitoring at the ICU.  ^c^Raised ICP as intracranial complication (requiring treatment) during ICU and/or hospital stay or, in case of brain specific monitoring at the ICU, controlled or refractory sustained raised ICP.  ^d^During ICU and/or hospital stay.  ^e^During ICU stay.  ^f^In or outside the hospital, up to 1 year after injury.  ^h^GOSE scores were assessed by in-person/telephonic interviews or postal questionnaires, and as such a clear distinction between GOSE 2 (vegetative state) and GOSE 3 (lower severe disability) was not always possible. As a result of this, these two categories were combined, giving a seven-point ordinal scale. When possible, missing values were imputed centrally from GOSE scores recorded at different time-points (2 weeks to one year after injury), using a multi-state model. | | | | | |
