# Supplementary material for: Clinical and Imaging Characteristics, Care Pathways, and Outcomes of Traumatic Epidural Hematomas: A Collaborative European NeuroTrauma Effectiveness Research in Traumatic Brain Injury Study
Source: Neurosurgery. 2024 May 21;95(5):986–99. doi: 10.1227/neu.0000000000002982 (PMC11449426; doi:10.1227/neu.0000000000002982)
Supplement: Supplementary file 4 [file neu-95-0986-s009.docx]

**Supplemental Digital Content 9, Table. Association of Baseline Clinical and Imaging Characteristics with Any Early Hematoma Evacuation**

| Characteristic | Descriptive statistics^a^ | | Unit for regression | Univariable  (n = 461) | Multivariable  (n = 461) | |
| --- | --- | --- | --- | --- | --- | --- |
|  | No AEHE (n=327) | AEHE (n=134) |  | OR (95% CI) | OR (95% CI) | R^2e^ |
| Age, median [IQR], years | 40  [24, 56] | 44  [28, 57] | per 10 years increase | 1.10 (0.90-1.22) | 1.00 (0.82-1.22) | 0.09 |
| Baseline GCS score, median [IQR] | 13 [8, 15] | 10 [4, 14] | per point increase, <9 | 1.05 (0.93-1.18) | 1.08 (0.89-1.32) |  |
|  |  |  | per point increase, ≥ 9 | **0.80 (0.71-0.90)** | 0.86 (0.71-1.05) |  |
| Baseline one/both unreactive pupils (%) | 37  (12.1) | 22 (17.3) | present | 1.28 (0.73-2.26) | 0.58 (0.18-1.90) |  |
| Focal neurologic deficit (%) | 26  (9.8) | 20 (21.1) | present | **2.00 (1.02-3.90)** | 0.96 (0.26-3.48) |  |
| Major extra-cranial injury (%) | 156 (47.7) | 59 (44.0) | present | 0.86 (0.57-1.29) | 1.81 (0.91-3.62) |  |
| EDH volume^b^, median [IQR], cm^3^ | 4  [1, 8] | 34  [13, 66] | per cm^3^ increase, < 30 cm^3^ | **1.12 (1.09-1.15)** | **1.14 (1.10-1.18)** | 0.52 |
|  |  |  | per cm^3^ increase, ≥ 30 cm^3^ | **1.02 (1.00-1.04)** | 1.01 (0.99-1.02) |  |
| Temporal EDH^c^ (%) | 173 (52.9) | 92 (68.7) | present | **1.95 (1.27-2.99)** | 1.57 (0.81-3.06) |  |
| ASDH^d^ (%) | 131 (40.1) | 66 (49.3) | present | 0.74 (0.43-1.27) | 0.70 (0.31-1.57) | 0.60 |
| ASDH volume^b^, median [IQR], cm^3^ | 0 [0, 3] | 0 [0, 14] | per cm^3^ increase, < 30 cm^3^ | **1.06 (1.03-1.10)** | **1.05 (1.00-1.11)** |  |
|  |  |  | per cm^3^ increase, ≥ 30 cm^3^ | 1.00 (0.98-1.02) | 1.00 (0.97-1.02) |  |
| IPH^d^ (%) | 195 (59.6) | 97 (72.4) | present | **1.70 (1.07-2.71)** | 1.12 (0.51-2.45) |  |
| IPH volume^b^, median [IQR], cm^3^ | 1 [0, 7] | 3 [0, 11] | per cm^3^ increase | 1.00 (0.99-1.01) | 1.00 (0.98-1.01) |  |
| TSAH (%) | 227 (69.4) | 106 (79.1) | present | **1.67 (1.03-2.69)** | 1.51 (0.65-3.50) |  |
| Midline shift (%) | 15  (4.6) | 80 (59.7) | present | **30.81 (16.51-57.52)** | **8.82 (3.47-22.45)** | 0.65 |
| Cisternal compression (%) | 66  (20.2) | 90  (67.2) | present | **8.09 (5.15-12.71)** | 1.78 (0.85-3.75) |  |
| *Abbreviations: AEHE, any early hematoma evacuation, regardless of which lesion was the main surgical indication; ASDH, acute subdural hematoma; EDH, epidural hematoma; GCS, Glasgow Coma Scale; IPH, intraparenchymal hemorrhage; IQR, interquartile range; TSAH, traumatic subarachnoid hemorrhage.*  ^a^Containing missing values for baseline GCS score, baseline pupils and focal neurologic deficit, as reported in Table 1. The univariable and multivariable regression models used imputed values.  ^b^Volumes of individual lesions were estimated using the width × depth × length × 0.5 formula. When multiple lesions of a given type were present simultaneously, their volumes were added up.  ^c^EDH extending into the temporal region (e.g., temporal, temporoparietal, temporofrontal) compared to EDH without extension into temporal region.  ^d^Binary indicator variables for the presence or absence of ASDH and IPH were included to adjust the respective continuous volume variables, which displayed spikes at zero, in both “univariable” and multivariable analysis.  ^e^Model Nagelkerke pseudo-R^2^s were calculated for models including the covariates on the corresponding rows and the rows above in the table. For example, the 0.52 pseudo-R^2^ is calculated for the model including age, GCS (piecewise), pupil reactivity, focal neurological deficit, major extra-cranial injury, EDH volume (piecewise) and temporal EDH. As more covariates are added to a model, the proportion of explained “variation” of the outcome, in this case whether early targeted EDH evacuation occurred, increases.  Pre-injury systemic disease (according to American Society of Anesthesiologists - Physical Status classification system) and admission stratum were considered as potential predictors. Neither significantly changed the association estimates of the other covariates in the full model, and the proportion of explained variance did not increase with the addition of either. In multivariable analysis, intensive care unit admission vs. ward admission was associated with any early hematoma evacuation (OR 3.81, 95% CI 1.14-12.79), but pre-injury systemic disease was not. | | | | | | |
